# Heterologous expression and purification of *Leptospira *spp recombinant proteins to leptospirosis vaccine development

**DOI:** 10.1186/1753-6561-8-S4-P141

**Published:** 2014-10-01

**Authors:** Ivânia Deliberalli, Everton Bettin, André Grassmann, Amilton Neto, Karina Colonetti, Odir Dellagostin, Everton Silva

**Affiliations:** 1 Laboratório de Vacinologia - Centro de Desenvolvimento Tecnológico - Unidade de Biotecnologia - UFPel, Pelotas, Brazil; 2 Grupo de Pesquisas em Doenças Transmitidas por Animais - Faculdade de Veterinária - UFPel, Pelotas, Brazil

## Background

Leptospirosis is an infectious disease of humans and other mammals and an important public health problem worldwide, mainly in developing countries. The disease is among the most common zoonosis worldwide and is caused by infection with pathogenic spirochetes of the genus *Leptospira*. [1]. Rodents and others wild or domestic animals are asymptomatic hosts that can carry *Leptospira *spp in the kidneys and shed high number of them in urine. The infection usually occurs through direct contact with reservoirs contaminated urine [1]. Leptospires have a double membrane structure, where the LPS are the main constituting antigen in its outer membrane. Those proteins are potential targets to development of vaccines due to its capability of being recognized by the hosts immune system [2-4]. There is no vaccine evaluable worldwide to protect humans against leptospirosis. In the last decades a number of studies evaluated recombinant proteins as vaccine antigens, with limited or no success. We randomly chose three conserved lipoproteins to evaluate as recombinant proteins: those expressed by lic10260, lic10365 and lic11360 ORFs in the *L. interrogans *L1-130 genome. Lipoproteins are associated with membranes and may be exposed in the cell surface, allowing recognition by immune responses. In this study we cloned, expressed and purified these proteins.

## Methods

Primers were designed to add restriction enzyme sites to each gene that were amplified from *Leptospira borgpetersenii sorovar *Ballum strain 4E genomic DNA. The sequences were cloned into *E. coli *pAE expression vector. The recombinant vectors were utilized to express recombinant *Leptospira *proteins in E. coli BL21 (DE3) *Star *cells. The cultures were grown to log phase and the recombinant proteins expression induced by adding IPTG. The cells were harvested, lysed and the proteins were purified in denaturing conditions using Ni^2+ ^affinity chromatography. Purified proteins were analyzed by SDS-PAGE and Western Blot with anti-6xHis antibody. The final concentration of each protein was determined using the commercial kit BCA Protein Assay.

## Results and conclusions

The cloning was successful and resulted in the pAE/lic10260, pAE/lic10365 and pAE/lic11360 recombinant vectors. The proteins were expressed in E. coli BL21 (DE3) Star and purified, yielding high amount each with satisfactory purity. The WB with anti-6xHis antibody marked recombinant proteins in the expected size: 12 kDa for rLIC10260; 37 kDa for rLIC10365 e 24 kDa for rLIC11360. These proteins are now target antigens to develop a recombinant vaccine against leptospirosis. Currently we are evaluating these proteins in an established hamster model of leptospirosis using homologous and heterologous challenges.
